# MRI Types of Cerebral Small Vessel Disease and Circulating Markers of Vascular Wall Damage

**DOI:** 10.3390/diagnostics10060354

**Published:** 2020-05-29

**Authors:** Larisa A. Dobrynina, Maryam R. Zabitova, Alla A. Shabalina, Elena I. Kremneva, Bulat M. Akhmetzyanov, Zukhra Sh. Gadzhieva, Alexander B. Berdalin, Ludmila A. Kalashnikova, Elena V. Gnedovskaya, Marina V. Krotenkova

**Affiliations:** 1 Research Center of Neurology, 80 Volokolamskoe shosse, 125367 Moscow, Russia; m_zabitova@mail.ru (M.R.Z.); ashabalina@yandex.ru (A.A.S.); kremneva@neurology.ru (E.I.K.); zuhradoc@mail.ru (Z.S.G.); kalashnikovancn@yandex.ru (L.A.K.); gnedovskaya@mail.ru (E.V.G.); krotenkova_mrt@mail.ru (M.V.K.); 2 LLC PET-Technology, 58, korpus 2, ulica Zorge, 450054 Ufa, Russia; doctorbulat@mail.ru; 3 Federal State Budgetary Institution “Federal Center for Cerebrovascular Pathology and Stroke”, 1, stroenie 10, Ostrovityanova, 117342 Moscow, Russia; alex_berdalin@mail.ru

**Keywords:** cerebral small vessel disease, neuroimaging, MRI-types, white matter hyperintensities, vascular endothelial growth factor A, tumor necrosis factor-alpha, cognitive dysfunction

## Abstract

The evaluation of the clustering of magnetic resonance imaging (MRI) signs into MRI types and their relationship with circulating markers of vascular wall damage were performed in 96 patients with cerebral small vessel disease (cSVD) (31 men and 65 women; mean age, 60.91 ± 6.57 years). The serum concentrations of the tumor necrosis factor-α (TNF-α), transforming growth factor-β1 (TGF-β1), vascular endothelial growth factor-A (VEGF-A), and hypoxia-inducible factor 1-α (HIF-1α) were investigated in 70 patients with Fazekas stages 2 and 3 of white matter hyperintensities (WMH) and 21 age- and sex-matched volunteers with normal brain MRI using ELISA. The cluster analysis excluded two patients from the further analysis due to restrictions in their scanning protocol. MRI signs of 94 patients were distributed into two clusters. In the first group there were 18 patients with Fazekas 3 stage WMH. The second group consisted of 76 patients with WMH of different stages. The uneven distribution of patients between clusters limited the subsequent steps of statistical analysis; therefore, a cluster comparison was performed in patients with Fazekas stage 3 WMH, designated as MRI type 1 and type 2 of Fazekas 3 stage. There were no differences in age, sex, degree of hypertension, or other risk factors. MRI type 1 had significantly more widespread WMH, lacunes in many areas, microbleeds, atrophy, severe cognitive and gait impairments, and was associated with downregulation of VEGF-A compared with MRI type 2. MRI type 2 had more severe deep WMH, lacunes in the white matter, no microbleeds or atrophy, and less severe clinical manifestations and was associated with upregulation of TNF-α compared with MRI type 1. The established differences reflect the pathogenetic heterogeneity of cSVD and explain the variations in the clinical manifestations observed in Fazekas stage 3 of this disease.

## 1. Introduction

Cerebral small vessel disease (cSVD), which is associated with age and vascular risk factors, is the leading cause of vascular and mixed dementia, stroke, gait, and urinary disturbances [1,2,3,4,5,6]. MRI signs are crucial for the diagnosis of cSVD and for evaluating its progression [4,7,8]. Recent studies have shown that the combination of signs and the overall burden of cSVD better predict the cognitive decline in this condition [9,10,11,12]. Concomitantly, patients with cSVD exhibit significant differences in the rate of disease progression [13] and the nature of brain damage [14,15], which can be explained by the heterogeneity of the disease and its pathogenesis. The pathophysiology of cSVD has not been completely established [8,16,17]. Therefore, it is especially important to improve the MRI-based diagnosis of cSVD and to study the mechanisms of its development [7,18,19,20], to identify the pathophysiological markers of disease progression, for example, by assessing the relationship between MRI data and the blood parameters associated with various mechanisms of vessel and brain damage [16,21,22,23]. We hypothesized that a sequential analysis of MRI signs severity in various parts of the brain and their combination will be able to reveal groups (clusters) of MRI signs, most likely because of differences in their pathogenesis. In this way, comparing clusters of MRI signs with circulating markers of vascular and cerebral damage can help to differentiate the pathogenetic variants of cSVD and the associated clinical manifestations. We studied tumor necrosis factor-α (TNF-α), transforming growth factor-β1 (TGF-β1), hypoxia-inducible factor 1-α (HIF-1α), and vascular endothelial growth factor-A (VEGF-A), which were linked with the presence of MRI signs of cSVD in a previous screening study of individuals of working age with and without hypertension [22]. Moreover, these factors were chosen because experimental evidence showed their involvement in the leading mechanisms of cSVD development—damage of the endothelium and extracellular matrix with subsequent remodeling of the vascular wall—as well as the role of chronic inflammation accompanied by high vascular wall permeability in initiating and maintaining these processes [24,25,26,27]. Few clinical studies investigated the significance of these circulating markers of vascular wall and cerebral damage. A link has been established between polymorphism of the promoter of the *TNF-α* gene and increased TNF-α levels, ischaemic stroke [28] and increased TGF-β1 levels with progressive deep white matter lesions in patients with cSVD [29]. Data on VEGF-A are inconsistent; its level showed a non-linear relationship with cardiovascular risk, as it was low in the lower and upper quartile values and high in the middle quartile values [30]. Finally, no link between VEGF-A levels and the severity of the MRI signs of cSVD was observed in middle-aged individuals [31], but was present in older patients [17].

### Aim

Our aim was to identify the MRI types of cSVD based on the grouping of MRI signs and their relationship with circulating markers of vascular wall damage.

## 2. Materials and Methods

We studied 96 patients with cSVD (31 men and 65 women; mean age, 60.91 ± 6.57 years).

### 2.1. Inclusion Criteria

The inclusion criteria of this study were an age of 46–70 years and brain MRI changes corresponding to cSVD (lacunes, white matter hyperintensities (WMH), enlarged perivascular spaces, microbleeds, and brain atrophy) [7]. Patients with Fazekas stage 1 WMH were included in the study if they had grade 2 or 3 hypertension and/or ≥1 lacunar infarction.

### 2.2. Exclusion Criteria

The exclusion criteria of this study were (1) severe dementia; (2) cognitive disorder likely caused by Alzheimer’s disease [32,33]; (3) small subcortical stroke/lacunar infarction <3 months after an acute cerebrovascular event; (4) cSVD caused by other, independent factors (genetic, inflammatory, thrombophilic, systemic, toxic, or severe migraine, as gleaned from the medical history); (5) presence of another cause of stroke and concomitant brain pathology other than cSVD; (6) >50% atherosclerosis of the extra- or intracranial arteries; (7) serious medical conditions—cardiac (ejection fraction <50%), endocrine (type 1 or 2 diabetes mellitus with serious vascular complications, uncompensated thyroid dysfunction), renal (chronic kidney disease with a glomerular filtration rate <30 mL/min) and others; and (8) contraindications for MRI studies.

The control group consisted of 21 healthy volunteers (6 men and 15 women; mean age, 57.33 ± 5.19 years) without clinical and MRI evidence of vascular and degenerative brain pathology.

The study was approved by the Local Ethics Committee of the Center of Neurology (Moscow, Russia). The ethics statement number is 2–4/16 dated 17 February 2016. All subjects signed an informed consent form for the study and processing of their personal data.

### 2.3. Clinical Evaluation

All patients were asked about the development of general and neurological disease symptoms. Their physical state, major vascular risk factors [34], and neurological syndromes were evaluated. The severity of cognitive impairment was assessed using both the Montreal Cognitive Assessment (MoCA) scale [35] and independence in everyday life [36]. Dementia was diagnosed if the patient scored ≤26 points on the MoCA and was dependent on others, whereas a score of ≤26 points and independence was classified as mild cognitive impairment, and a score of >26 points and the presence of cognitive complaints was classified as subjective cognitive impairment. Gait disturbances unrelated to hemiparesis were rated according to severity: mild—altered gait only during difficult tests (tandem, sideways walking); moderate—reduced step length, reduced gait speed, instability during difficult tests without changes in the base of support, gait rhythm, or need for support; significant—clearly evident frontal cerebellar or frontal subcortical types of gait disturbances, without support or with intermittent unilateral support; and severe—presence of frontal cerebellar or frontal subcortical types of gait disorders with the need for unilateral or bilateral support.

### 2.4. MRI Study and Data Analysis

An MRI study and data analysis were performed in all patients. MRI data were acquired using a Siemens MAGNETOM Verio 3T scanner (Siemens Medical Systems, Erlangen, Germany) with a standard 12-channel matrix head coil. To evaluate the STandards for ReportIng Vascular changes on nEuroimaging (STRIVE) criteria [7], patients and the control group underwent axial spin-echo T2-weighted imaging (TR, 4000 ms; TE, 118 ms; slice thickness, 5.0 mm; in-plane resolution, 1.5 mm^2^; duration, 2 min 02 s); sagittal 3D T2 FLAIR (TR, 6000 ms; TE, 395 ms; isotropic voxel 1 × 1 × 1 mm; duration, 7 min 12 s); sagittal 3D T1-mpr (TR, 1900 ms; TE, 2.5 ms; isotropic voxel, 1 × 1 × 1 mm; duration, 4 min 16 s; diffusion MRI (DWI) using an axial spin-echo echo-planar imaging sequence with two b-values (0, 1000 s/mm^2^) (TR, 4000 ms; TE, 100 ms; slice thickness, 4 mm; duration, 1 min 20 s); and axial susceptibility weighted imaging sequence (SWI) with magnitude and phase image reconstruction (TR, 28 ms; TE, 20 ms; slice thickness, 1.2 mm; FOV, 179 × 230 mm; duration, 8 min 12 s) was performed.

Two neuroradiologists (E.K. and B.A.) evaluated the brain MRI studies in a standardized manner while being blinded to clinical information. No STRIVE criteria were found in the control group. MRI signs were evaluated according to severity in various brain regions, for subsequent cluster analysis (Tables S1–S4). Lacunes were evaluated on T2 FLAIR images separately in the white matter, basal ganglia, cerebellum, and brainstem, depending on their amount: none, <5, 5–10, or >10. WMH were evaluated on T2 FLAIR images using the Fazekas scale (grade 0–3) and on a 4-point severity scale in the anterior and posterior regions of the frontal, parietal, temporal, and occipital lobes in the juxtacortical (up to 4 mm from the border between the cerebral cortex and white matter), periventricular (up to 13 mm from the lateral ventricles), and deep (zone between periventricular and deep white matter) white matter, separately [37]. Microbleeds were rated on SWI images depending on their number (<5, 5–10, and >10); microbleeds in the basal ganglia and frontal, parietal, occipital, and temporal lobes were calculated separately. Perivascular spaces were graded based on size (1–4 mm) in the centrum semiovale and basal ganglia. Cerebral atrophy was evaluated based on the severity of subarachnoid enlargement in separate lobes of the brain [38].

### 2.5. Blood Parameters

The study of blood parameters was conducted in 70 patients (23 men and 47 women; mean age, 60.1 ± 6.5 years) and 21 volunteers (6 men and 15 women; mean age, 57.33 ± 5.19 years).

The levels of VEGF-A (Kit BCM Diagnostic, Woodland, CA, USA), TNF-α (Kit BCM Diagnostic, Woodland, CA, USA), HIF-1α (Kit Cusabio, Houston, TX, USA), and TGF-β1 (Kit Cusabio, Houston, TX, USA) were determined by ELISA using a VICTOR 2 plate reader (Perkin Elmer, Waltham, MA, USA). Venous blood was collected in the morning in a fasting state and placed in Vacutainer tubes (Greiner Bio-One, Kremsmünster, Austria) with a coagulation activator to obtain serum. Quality control of the parameters measured by the ELISA method was performed in duplicates using lyophilized control sera with a low and high concentration of the studied analytes.

### 2.6. Statistical Analysis

Statistical analysis was performed using the IBM SPSS 23.0 software (IBM SPSS Statistics, version 23.0, IBM Corp., Armonk, NY, USA) and R 3.4.3. (R Foundation for Statistical Computing, Vienna, Austria). Frequency and percentage (%) were used as descriptive statistics for categorical and ordinal variables, respectively, and median and quartiles were used for quantitative variables. In all cases, two-way versions of the statistical criteria were used. The null hypothesis was rejected if *p* < 0.05.

The frequencies of qualitative variable categories according to independent (grouping) variable levels were compared using the χ^2^ test or Fisher’s exact test.

A cluster analysis using the agglomerative hierarchical method and iterative k-means algorithm was performed on the severity of the MRI signs of cSVD in different parts of the brain [39]. This method allowed us to identify and group repeated change patterns across multiple variables. The problem of non-homogeneity of the measurement units was solved by using preliminary standardization of variables and calculating the standardized score (Z-score).

The quantitative variables according to independent (grouping) variable levels were compared using the Kruskal–Wallis test, followed by the Mann–Whitney *U* test with Bonferroni correction. Spearman’s correlation analysis was used to assess the correlation between quantitative variables. To determine the effect of vascular risk factors on the levels of VEGF-A, TNF-α, HIF-1α, and TGF-β1, a multiple linear regression analysis was performed with stepwise model selection.

## 3. Results

Table 1 shows the general characteristics of the patients with cSVD and the control group.

The study groups were similar regarding sex and age. Hypertension was more severe in 83% of the patients with cSVD compared with the control group.

Table 2 shows the features of the main clinical syndromes and MRI signs in patients with cSVD.

**Figure 1:**
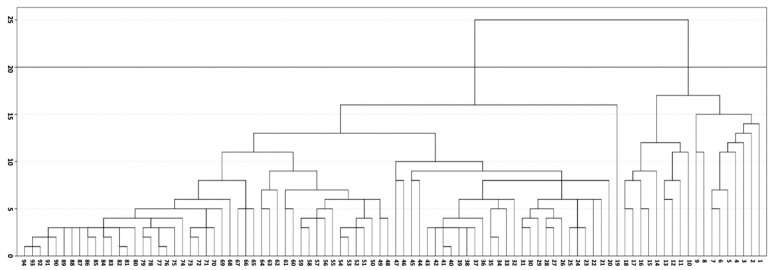
Dendrogram showing the hierarchical classification of patients. The *x*-axis represents the grouped objects; the *y*-axis is the cluster proximity.

MRI data on the severity of the MRI signs of cSVD in different parts of the brain (Tables S1–S4) were used for a hierarchical cluster analysis of the MRI signs of cSVD. The results are presented in a dendrogram (Figure 1), which displays all clusters obtained and their nesting relative to each other. We used an iterative k-means method that minimized the total square deviation of cluster points from the center of these clusters and selected clusters based on visual analysis. In Figure 1, the horizontal line cuts through the dendrogram at the level of maximum cluster separation, visually highlighting the two clusters.

Two patients could not be assigned to any of the clusters because of restrictions in their scanning protocol. The first cluster included 18 patients with Fazekas stage 3 WMH; the second cluster included 76 patients with different stages of WMH (22 with stage 3, 28 with stage 2, and 26 with stage 1). The uneven distribution of patients between clusters limited the subsequent steps of statistical analysis; therefore, a cluster comparison was performed in patients with Fazekas stage 3 WMH. The clusters were designated as MRI type 1 or type 2, Fazekas stage 3. MRI type 1 (*n* = 18; 6 women; mean age, 59.1 ± 6.8 years) and type 2 (*n* = 22; 15 women; mean age, 63.5 ± 6.2 years) did not differ in age, sex, degree of hypertension, or presence of other risk factors.

**Figure 2:**
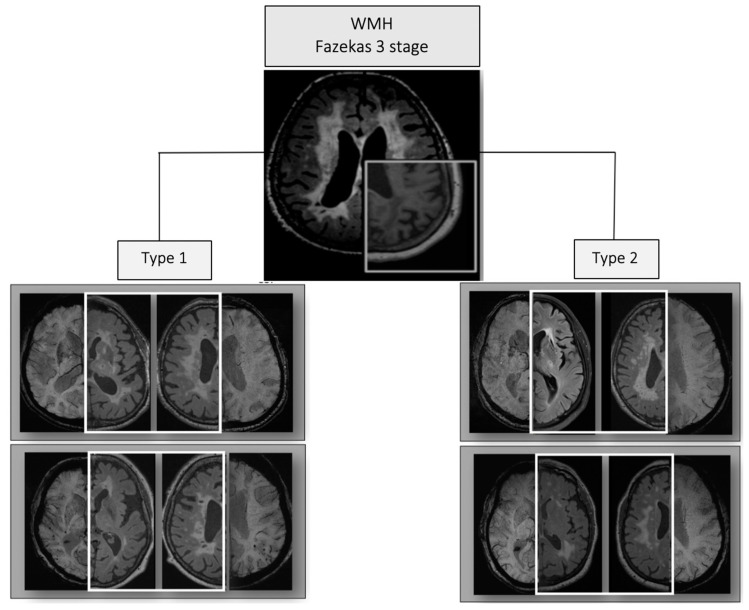
Characteristic changes observed in MRI types 1 and 2 of Fazekas stage 3 cerebral small vessel disease (cSVD) on FLAIR (highlighted with a white square) and axial susceptibility weighted imaging sequence (SWI) MRI slices.

MRI type 1 of Fazekas stage 3 cSVD exhibited a significantly increased (*p* < 0.05) prevalence of periventricular WMH in all parts of the cerebral hemispheres, brainstem, and subcortical structures; number of lacunes and microbleeds in all regions; and atrophy compared with MRI type 2. MRI type 2 of Fazekas stage 3 cSVD displayed a greater periventricular temporoparietal or juxtacortical and deep WMH, lacunes in the cerebral white matter and enlarged perivascular spaces around the basal ganglia, in the absence of microbleeds (Figure 2).

Patients with MRI type 1 of Fazekas stage 3 cSVD had a more severe cognitive impairment (*p* = 0.006) and gait disturbances unrelated to hemiparesis (*p* = 0.01) compared with MRI type 2.

**Figure 3:**
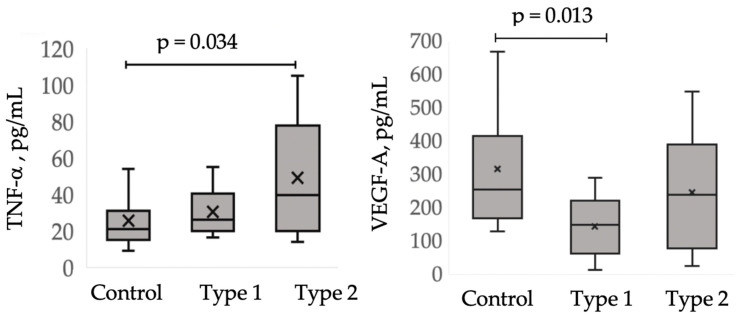
Comparative analysis of vascular endothelial growth factor-A (VEGF-A) and tumor necrosis factor-α (TNF-α) levels between patients with MRI type 1 and MRI type 2 and the controls.

A one-way analysis of the Kruskal–Wallis test revealed an association of the MRI types of Fazekas stage 3 cSVD with the level of TNF-α (*p* = 0.039) and VEGF-A (*p* = 0.016), but not with TGF-β1 (*p* = 0.141) or HIF-1α (*p* = 0.110). The comparison of these blood parameters between patients with MRI type 1, those with MRI type 2, and the control group are shown in Figure 3, highlighting significant differences. Compared with the control group, patients with MRI type 1 were characterized by a decrease in VEGF-A levels (*p* = 0.013), whereas patients with MRI type 2 were characterized by an increase in TNF-α levels (*p* = 0.034). The levels of TNF-α and VEGF-A exhibited a tendency to be different between MRI types 1 and 2.

Correlations were established between the levels of TNF-α and TGF-β1 (*r* = 0.378, *p* = 0.000), TNF-α and HIF-1α (*r* = 0.220, *p* = 0.035), and VEGF-A and HIF-1α (*r* = 0.237, *p* = 0.023).

## 4. Discussion

The role of MRI signs in the diagnosis of cSVD [7,8], as well as the advantages of evaluating a combination of MRI signs to characterize brain damage, clinical symptoms [9,10,11,12], differences in the type of brain damage [14,15], and cSVD progression [13], were the basis of our hypothesis of grouping the MRI signs of cSVD according to this pathogenetic homogeneity. The severity of MRI signs in different parts of the brain was evaluated on a point scale, and their co-occurrence was verified using a cluster analysis. Regions of white matter were divided according to their blood supply [40,41] and the classical division of the brain into lobes was employed, which generally matched the currently used anatomical division of white matter in the elderly [37]. The identified clusters of Fazekas stage 3 MRI signs, designated as MRI type 1 and type 2, exhibited significant differences in their neuroimaging profiles (Figure 2). The significant variability in the severity of clinical symptoms, despite a lack of differences in the main risk factors for cSVD (i.e., hypertension and age), served as evidence that these MRI types are associated with a predominance of certain pathological mechanisms and are not stages of the disease. Consequently, the characteristic variation in the levels of circulating blood markers found in MRI type 1 and type 2 may indicate the significance of the associated mechanisms in vascular and brain damage.

The decrease in the level of VEGF-A, which was typical of MRI type 1, was most likely associated with serious and widespread vascular wall damage, with disturbances in the autocrine synthesis of VEGF-A by endothelial cells and their death [42]. This is consistent with the more pronounced MRI changes and clinical manifestations observed for MRI type 1 compared with type 2. The possibility of such an outcome has been morphologically confirmed [43]. VEGF-A is a major factor in angiogenesis and regulates, among other processes, the proliferation, survival, and migration of endothelial cells, as well as vascular permeability [44]. The data obtained here regarding a link between the decrease in VEGF-A and the formation of a specific neuroimaging profile may explain the contradictory relationship reported between VEGF-A and cSVD [17,30,31,45,46]. Most likely, the lack of change in VEGF-A level in patients with MRI type 2 compared with the controls indicates sufficient endothelial preservation, which is supported by the milder neuroimaging and clinical signs of this group. Our study detected a direct correlation between VEGF-A and HIF-1α (*r* = 0.237, *p* = 0.023). This is consistent with the established role of HIF-1A, which is a key transcription factor in hypoxia [47], in VEGF-mediated disturbances in BBB integrity and increased permeability [48,49], and of this mechanism as a whole in the development of cSVD [25,50].

The increase in TNF-α detected in patients with MRI type 2 demonstrates the significance of this major systemic pro-inflammatory cytokine [51,52,53,54] in vascular and cerebral damage, with formation of typical MRI changes. An increase in TNF-α levels was associated with increased BBB permeability, tissue oedema, inflammation, oligodendrocyte death, and destruction of the myelin basic protein [55,56,57,58,59]. TNF-R1 activation was associated with pro-inflammatory, cytotoxic, and apoptotic responses involving NF-κB and protein kinase, whereas TNF-R2 was associated with cell activation, proliferation and migration [53,54]. Similar to other cytokines, TNF-α can pass through an intact BBB by transcytosis [60]. The established role of TNF-α-mediated reactions in the formation of type 2 MRI changes is consistent with research data showing that white matter damage is not just a consequence of chronic hypoxia, but is caused and maintained by a pro-inflammatory environment [61]. Previously, we established an association between TNF-α and WMH in younger patients with signs of cSVD on MRI, which was unrelated to hypertension, leading to the hypothesis that TNF-α-mediated reactions play an independent role in the development of early-onset cSVD [22]. The potential link with MRI type 2 in the subsequent development of mixed dementia cannot be ruled out. The MRI changes detected in MRI type 2 vs. MRI type 1 cSVD included less damage to the lenticulostriate arteries and the anterior parts of the brain, which is typical in classic hypertensive cSVD with ischemic subcortical lesions [1]. TNF-α-mediated inflammation is recognized as a significant mechanism of AD induction, and anti-TNF therapy has been shown to decrease the pathological brain changes in rodent models of AD, as well as to slow down cognitive decline and improve everyday function in patients with AD [62]. The direct correlation between TNF-α and TGF-β1 (*r* = 0.378, *p* = 0.000) may indicate the interdependence of processes involving these circulating markers. The TGF-β signaling pathway is common in the pathogenesis of cSVD and AD [63,64] and may be one of the links that mediates the development of mixed cognitive impairment and degeneration. Moreover, TGF-β overexpression in rodent astrocytes caused small vessel changes similar to those found in patients with AD [65]. These changes were characterized by basal-membrane thickening, capillary degeneration, and the formation of string vessels [66]. TGF-β1 was involved in cSVD mechanisms, such as BBB damage [67] and increased artery stiffness and remodeling [68,69,70], and the development of progressive deep white matter lesions in patients with cSVD [29]. Interestingly, the level of HIF-1A was correlated significantly with biomarkers in both MRI types—VEGF-A in type 1 and TNF-α in type 2—which confirms the universal role of hypoxia in initiating and maintaining vascular wall damage in cSVD [25].

Thus, assessing the severity of MRI signs in different parts of the brain and grouping them into sign clusters allowed us to identify the MRI types of Fazekas stage 3 cSVD, which are thought to be associated with the predominance of different mechanisms of vascular and cerebral damage. The MRI types differed in the level of circulating markers of vascular wall damage and clinical signs of cSVD, which demonstrates the pathogenetic heterogeneity of cSVD and explains the differences in cognitive impairment and gait disturbances observed at Fazekas stage 3 in different patients. MRI type 1 had more severe clinical symptoms, such as widespread WMH (including in periventricular areas), lacunes, microbleeds, and atrophy, and was associated with VEGF-A downregulation, which may correspond to severe damage of the vascular wall and endothelial death. MRI type 2 cSVD exhibited milder clinical symptoms, mainly widespread juxtacortical and deep-brain WMH, white matter lacunes, absence of microbleeds, or atrophy, and was associated with TNF-α upregulation, which suggests that inflammation plays a dominant role in its formation, with an increase in BBB permeability and vascular wall remodeling.

In conclusion, this study established the diagnostic and pathogenetic value of differentiating the MRI types of Fazekas stage 3 cSVD. These findings support the need to improve the diagnostic criteria of these types and to clarify the leading mechanisms underlying their formation, to develop pathogenetically justified prevention and treatment strategies.

## Figures and Tables

**Table 1:** General characteristics of the patients with cerebral small vessel disease and the control group.

Parameter	cSVD (*n*, %)	Control (*n*, %)	*p*
Age (years)	60.91 ± 6.57	59.13 ± 6.56	0.615
Sex, females	64 (66.7%)	15 (65.2%)	0.814
Hypertension degree	82 (85.4%)	10 (43.5%)	**0.001**
1	9 (9.4%)	5 (21.7%)	
2	20 (20.8%)	4 (17.4%)	
3	53 (55.2%)	1 (4.3%)	
Diabetes mellitus	17 (17.7%)	0 (0%)	**0.022**
Smoking	25 (26%)	7 (30.4%)	0.440
Body mass index	28.85 ± 4.28	27.91 ± 4.32	0.617

The bold text indicates statistically significant differences.

**Table 2:** Features of the main clinical syndromes and MRI signs in patients with cerebral small vessel disease.

Signs	Patients with cSVD, *n* (%)
Cognitive impairment:	96 (100%)
dementia	15 (15.6%)
mild cognitive impairment	46 (47.9%)
subjective cognitive impairment	35 (36.5%)
Gait disorders not related to hemiparesis:	51 (53.2%)
mild	26 (27.1%)
moderate	11 (11.5%)
significant	12 (12.5%)
severe	2 (2.1%)
Urinary disturbances:	35 (36.5%)
urinary frequency	20 (20.8%)
urinary incontinence	15 (15.6%)
White matter hyperintensities (stages): F1/F2/F3	26 (27.1%)/31 (32.3%)/39 (40.6%)
Lacunes (*n*):	
in basal ganglia: none/<5/5–10/>10	32 (33.3%)
in cerebral white matter: none/<5/5–10/>10	42 (43.8%)
Microbleeds (*n*)	
in basal ganglia: none/<5/5–10/>10	28 (29.2%)
in temporal lobes: none/<5/5–10/>10*	24 (25%)
Enlarged perivascular spaces (mm):	
in basal ganglia: 3/4 mm (single)	28 (29.2%)
in the centrum semiovale: 3/4 mm	4 (4.2%)
Widening of the subarachnoid spaces in the temporal lobes	15 (15.6%)

* In 70% of patients, multiple microbleeds (>10) were detected in both the temporal lobe white matter and the subcortical structures.

## Supplementary Materials

Supplementary materials can be found at https://www.mdpi.com/2075-4418/10/6/354/s1.

## Abbreviations

cSVD	Cerebral small vessel disease
MRI	Magnetic resonance imaging
TGF-β1	Transforming growth factor-β1
TNF-α	Tumor necrosis factor-α
VEGF-A	Vascular endothelial growth factor-A
WMH	White matter hyperintensities
STRIVE	STandards for ReportIng Vascular changes on nEuroimaging

## References

[1] Pantoni L., Gorelick P.B. (2014). Cerebral Small Vessel Disease.

[2] Deramecourt V., Slade J.Y., Oakley A.E., Perry R.H., Ince P.G., Maurage C.A., Kalaria R.N. (2012). Staging and natural history of cerebrovascular pathology in dementia. Neurology.

[3] Poggesi A., Pantoni L., Inzitari D., Fazekas F., Ferro J., O’Brien J., Hennerici M., Scheltens P., Erkinjuntti T., The LADIS Study Group (2011). 2001–2011: A Decade of the LADIS (Leukoaraiosis And DISability) study: What have we learned about white matter changes and small-vessel disease?. Cereb. Dis..

[4] Pantoni L., Fierini F., Poggesi A., The LADIS Study Group (2015). Impact of cerebral white matter changes on functionality in older adults: An overview of the LADIS study results and future directions. Geriatr. Gerontol. Int..

[5] Pinter D., Ritchie S.J., Doubal F., Gattringer T., Morris Z., Bastin M.E., Del C.V.H.M., Royle N.A., Corley J., Munoz Maniega S. (2017). Impact of small vessel disease in the brain on gait and balance. Sci. Rep..

[6] Banerjee G., Jang H., Kim H.J., Kim S.T., Kim J.S., Lee J.H., Im K., Kwon H., Lee J.M., Na D.L. (2018). Total MRI small vessel disease burden correlates with cognitive performance, cortical atrophy, and network measures in a memory clinic population. J. Alzheimers Dis..

[7] Wardlaw J.M., Smith E.E., Biessels G.J., Cordonnier C., Fazekas F., Frayne R., Lindley R.I., O’Brien J.T., Barkhof F., Benavente O.R. (2013). Neuroimaging standards for research into small vessel disease and its contribution to ageing and neurodegeneration. Lancet Neurol..

[8] Wardlaw J.M., Smith C., Dichgans M. (2013). Mechanisms of sporadic cerebral small vessel disease: Insights from neuroimaging. Lancet Neurol..

[9] Staals J., Makin S.D., Doubal F.N., Dennis M.S., Wardlaw J.M. (2014). Stroke subtype, vascular risk factors, and total MRI brain small-vessel disease burden. Neurology.

[10] Staals J., Booth T., Morris Z., Bastin M.E., Gow A.J., Corley J., Redmond P., Starr J.M., Deary I.J., Wardlaw J.M. (2015). Total MRI load of cerebral small vessel disease and cognitive ability in older people. Neurobiol. Aging..

[11] Arba F., Mair G., Carpenter T., Sakka E., Sandercock P.A.G., Lindley R.I., Inzitari D., Wardlaw J.M., Collaborators I.S.T.T. (2017). cerebral white matter hypoperfusion increases with small-vessel disease burden. Data from the third international stroke trial. J Stroke Cereb. Dis..

[12] Huijts M., Duits A., van Oostenbrugge R.J., Kroon A.A., de Leeuw P.W., Staals J. (2013). Accumulation of MRI markers of cerebral small vessel disease is associated with decreased cognitive function. A study in first-ever lacunar stroke and hypertensive patients. Front. Aging. Neurosci..

[13] Lawrence A.J., Brookes R.L., Zeestraten E.A., Barrick T.R., Morris R.G., Markus H.S. (2015). Pattern and rate of cognitive decline in cerebral small vessel disease: A prospective study. PLoS ONE.

[14] Schmidt R., Schmidt H., Haybaeck J., Loitfelder M., Weis S., Cavalieri M., Seiler S., Enzinger C., Ropele S., Erkinjuntti T. (2011). Heterogeneity in age-related white matter changes. Acta Neuropathol..

[15] Gouw A.A., Seewann A., van der Flier W.M., Barkhof F., Rozemuller A.M., Scheltens P., Geurts J.J. (2011). Heterogeneity of small vessel disease: A systematic review of MRI and histopathology correlations. J. Neurol. Neurosurg. Psychiatry.

[16] Poggesi A., Pasi M., Pescini F., Pantoni L., Inzitari D. (2016). Circulating biologic markers of endothelial dysfunction in cerebral small vessel disease: A review. J. Cereb. Blood Flow Metab..

[17] Arba F., Giannini A., Piccardi B., Biagini S., Palumbo V., Giusti B., Nencini P., Maria Gori A., Nesi M., Pracucci G. (2019). Small vessel disease and biomarkers of endothelial dysfunction after ischaemic stroke. Eur. Stroke J..

[18] Dichgans M., Wardlaw J., Smith E., Zietemann V., Seshadri S., Sachdev P., Biessels G.J., Fazekas F., Benavente O., Pantoni L. (2016). METACOHORTS for the study of vascular disease and its contribution to cognitive decline and neurodegeneration: An initiative of the joint programme for neurodegenerative disease research. Alzheimers Dement..

[19] Smith E.E., Biessels G.J., De Guio F., de Leeuw F.E., Duchesne S., During M., Frayne R., Ikram M.A., Jouvent E., MacIntosh B.J. (2019). Harmonizing brain magnetic resonance imaging methods for vascular contributions to neurodegeneration. Alzheimers Dement..

[20] Blair G.W., Hernandez M.V., Thrippleton M.J., Doubal F.N., Wardlaw J.M. (2017). Advanced neuroimaging of cerebral small vessel disease. Curr. Treat. Options Cardiovasc. Med..

[21] Shoamanesh A., Preis S.R., Beiser A.S., Vasan R.S., Benjamin E.J., Kase C.S., Wolf P.A., DeCarli C., Romero J.R., Seshadri S. (2015). Inflammatory biomarkers, cerebral microbleeds, and small vessel disease: Framingham Heart Study. Neurology.

[22] Dobrynina L.A., Gnedovskaya E.V., Shabalina A.A., Sergeeva A.N., Kravchenko M.A., Nikolaeva N.S. (2018). Biomarkers and mechanisms of early vascular damage. Zh. Nevrol. Psikhiatr. Im. SS Korsakova.

[23] Dobrynina L.A., Shabalina A.A., Zabitova M.R., Kremneva E.I., Gadzhieva Z.S., Krotenkova M.V., Gnedovskaya E.V., Berdalin A.B., Kalashnikova L.A. (2019). Tissue plasminogen activator and MRI signs of cerebral small vessel disease. Brain Sci..

[24] Wardlaw J.M., Makin S.J., Hernández M.C.V., Armitage P.A., Heye A.K., Chappell F.M., Thrippleton M.J. (2017). Blood-brain barrier failure as a core mechanism in cerebral small vessel disease and dementia: Evidence from a cohort study. Alzheimers Dement..

[25] Rosenberg G.A. (2018). Binswanger’s disease: Biomarkers in the inflammatory form of vascular cognitive impairment and dementia. J. Neurochem..

[26] Rajani R.M., Quick S., Ruigrok S.R., Graham D., Harris S.E., Verhaaren B.F.J., Fornage M., Seshadri S., Atanur S.S., Dominiczak A.F. (2018). Reversal of endothelial dysfunction reduces white matter vulnerability in cerebral small vessel disease in rats. Sci. Transl. Med..

[27] Low A., Mak E., Rowe J.B., Markus H.S., O’Brien J.T. (2019). Inflammation and cerebral small vessel disease: A systematic review. Ageing Res Rev..

[28] Cui G., Wang H., Li R., Zhang L., Li Z., Wang Y., Hui R., Ding H., Wang D.W. (2012). Polymorphism of tumor necrosis factor alpha (TNF-alpha) gene promoter, circulating TNF-alpha level, and cardiovascular risk factor for ischemic stroke. J. Neuroinflamm..

[29] Kuriyama N., Mizuno T., Kita M., Yamada K., Ozaki E., Matsumoto S., Takada A., Watanabe A., Kasai T., Nagakane Y. (2014). TGF-beta1 is associated with the progression of intracranial deep white matter lesions: A pilot study with 5 years of magnetic resonance imaging follow-up. Neurol. Res..

[30] Kaess B.M., Preis S.R., Beiser A., Sawyer D.B., Chen T.C., Seshadri S., Vasan R.S. (2016). Circulating vascular endothelial growth factor and the risk of cardiovascular events. Heart.

[31] Raman M.R., Himali J.J., Conner S.C., DeCarli C., Vasan R.S., Beiser A.S., Seshadri S., Maillard P., Satizabal C.L. (2018). Circulating vascular growth factors and magnetic resonance imaging markers of small vessel disease and atrophy in middle-aged adults. Stroke.

[32] Albert M.S., DeKosky S.T., Dickson D., Dubois B., Feldman H.H., Fox N.C., Gamst A., Holtzman D.M., Jagust W.J., Petersen R.C. (2011). The diagnosis of mild cognitive impairment due to Alzheimer’s disease: Recommendations from the national institute on aging-Alzheimer’s association workgroups on diagnostic guidelines for Alzheimer’s disease. Alzheimers Dement..

[33] McKhann G.M., Knopman D.S., Chertkow H., Hyman B.T., Jack C.R., Kawas C.H., Klunk W.E., Koroshetz W.J., Manly J.J., Mayeux R. (2011). The diagnosis of dementia due to Alzheimer’s disease: Recommendations from the national institute on aging-Alzheimer’s association workgroups on diagnostic guidelines for Alzheimer’s disease. Alzheimers Dement..

[34] Mancia G., Fagard R., Narkiewicz K., Redon J., Zanchetti A., Bohm M., Christiaens T., Cifkova R., De Backer G., Dominiczak A. (2013). 2013 ESH/ESC Guidelines for the management of arterial hypertension: The task force for the management of arterial hypertension of the European society of hypertension (ESH) and of the European society of cardiology (ESC). J. Hypertens..

[35] Nasreddine Z.S., Phillips N.A., Bédirian V., Charbonneau S., Whitehead V., Collin I., Cummings J.L., Chertkow H. (2005). The Montreal cognitive assessment, MoCA: A brief screening tool for mild cognitive impairment. J. Am. Geriatr Soc..

[36] American Psychiatric Association (2013). Diagnostic and Statistical Manual of Mental Disorders.

[37] Kim K.W., MacFall J.R., Payne M.E. (2008). Classification of white matter lesions on magnetic resonance imaging in elderly persons. Biol. Psychiatry.

[38] Pasquier F., Leys D., Weerts J.G., Mounier-Vehier F., Barkhof F., Scheltens P. (1996). Inter-and intraobserver reproducibility of cerebral atrophy assessment on MRI scans with hemispheric infarcts. Eur. Neurol..

[39] Duran B.S., Odell P.L. (2013). Cluster Analysis: A Survey.

[40] Rowbotham G.F., Little E. (1965). Circulations of the cerebral hemispheres. Br. J. Surg..

[41] Ravens J.R., Toole J.F., Hasegawa T. (1968). Anastomoses in the vascular bed of the human cerebrum. J. Neuropathol. Exp. Neurol..

[42] Lee S., Chen T.T., Barber C.L., Jordan M.C., Murdock J., Desai S., Ferrara N., Nagy A., Roos K.P., Iruela-Arispe M.L. (2007). Autocrine VEGF signaling is required for vascular homeostasis. Cell.

[43] Brown W.R., Thore C.R. (2011). Review: Cerebral microvascular pathology in ageing and neurodegeneration. Neuropathol. Appl. Neurobiol..

[44] Peach C.J., Mignone V.W., Arruda M.A., Alcobia D.C., Hill S.J., Kilpatrick L.E., Woolard J. (2018). Molecular pharmacology of VEGF-A isoforms: Binding and signalling at VEGFR2. Int J. Mol. Sci..

[45] Kwon H.S., Kim Y.S., Park H.H., Choi H., Lee K.Y., Lee Y.J., Heo S.H., Chang D.I., Koh S.H. (2015). Increased VEGF and decreased SDF-1alpha in patients with silent brain infarction are associated with better prognosis after first-ever acute lacunar stroke. J. Stroke Cerebrovasc Dis..

[46] Pikula A., Beiser A.S., Chen T.C., Preis S.R., Vorgias D., DeCarli C., Au R., Kelly-Hayes M., Kase C.S., Wolf P.A. (2013). Serum brain-derived neurotrophic factor and vascular endothelial growth factor levels are associated with risk of stroke and vascular brain injury: Framingham study. Stroke.

[47] Sharp F.R., Bernaudin M. (2004). HIF1 and oxygen sensing in the brain. Nat. Rev. Neurosci..

[48] Yeh W.L., Lu D.Y., Lin C.J., Liou H.C., Fu W.M. (2007). Inhibition of hypoxia-induced increase of blood-brain barrier permeability by YC-1 through the antagonism of HIF-1alpha accumulation and VEGF expression. Mol. Pharmacol..

[49] Zhang Z., Yan J., Shi H. (2016). Role of hypoxia inducible factor 1 in hyperglycemia-exacerbated blood-brain barrier disruption in ischemic stroke. Neurobiol. Dis..

[50] Fernando M.S., Simpson J.E., Matthews F., Brayne C., Lewis C.E., Barber R., Kalaria R.N., Forster G., Esteves F., Wharton S.B. (2006). White matter lesions in an unselected cohort of the elderly: Molecular pathology suggests origin from chronic hypoperfusion injury. Stroke.

[51] Gruys E., Toussaint M.J., Niewold T.A., Koopmans S.J. (2005). Acute phase reaction and acute phase proteins. J. Zhejiang Univ. Sci. B.

[52] Bradley J.R. (2008). TNF-mediated inflammatory disease. J. Pathol..

[53] Kollias G., Sfikakis P.P. (2010). TNF pathophysiology molecular and cellular mechanisms. Curr. Dir. Autoimmun..

[54] Apostolaki M., Armaka M., Victoratos P., Kollias G. (2010). Cellular mechanisms of TNF function in models of inflammation and autoimmunity. Curr. Dir. Autoimmun..

[55] Masumura M., Hata R., Nagai Y., Sawada T. (2001). Oligodendroglial cell death with DNA fragmentation in the white matter under chronic cerebral hypoperfusion: Comparison between normotensive and spontaneously hypertensive rats. Neurosci. Res..

[56] Didier N., Romero I.A., Creminon C., Wijkhuisen A., Grassi J., Mabondzo A. (2003). Secretion of interleukin-1beta by astrocytes mediates endothelin-1 and tumour necrosis factor-alpha effects on human brain microvascular endothelial cell permeability. J. Neurochem..

[57] Walker E.J., Rosenberg G.A. (2010). Divergent role for MMP-2 in myelin breakdown and oligodendrocyte death following transient global ischemia. J. Neurosci. Res..

[58] Yang Y., Jalal F.Y., Thompson J.F., Walker E.J., Candelario-Jalil E., Li L., Reichard R.R., Ben C., Sang Q.X., Cunningham L.A. (2011). Tissue inhibitor of metalloproteinases-3 mediates the death of immature oligodendrocytes via TNF-alpha/TACE in focal cerebral ischemia in mice. J. Neuroinflamm..

[59] Sawant D.A., Wilson R.L., Tharakan B., Stagg H.W., Hunter F.A., Childs E.W. (2014). Tumor necrosis factor-alpha-induced microvascular endothelial cell hyperpermeability: Role of intrinsic apoptotic signaling. J. Physiol. Biochem..

[60] Pan W., Kastin A.J. (2002). TNF alpha transport across the blood-brain barrier is abolished in receptor knockout mice. Exp. Neurol..

[61] Rouhl R.P., Damoiseaux J.G., Lodder J., Theunissen R.O., Knottnerus I.L., Staals J., Henskens L.H., Kroon A.A., de Leeuw P.W., Tervaert J.W. (2012). Vascular inflammation in cerebral small vessel disease. Neurobiol. Aging..

[62] Decourt B., Lahiri D.K., Sabbagh M.N. (2017). Targeting tumor necrosis factor alpha for Alzheimer’s disease. Curr. Alzheimer Res..

[63] Haffner C., Malik R., Dichgans M. (2016). Genetic factors in cerebral small vessel disease and their impact on stroke and dementia. J. Cereb. Blood Flow Metab..

[64] Thompson C.S., Hakim A.M. (2009). Living beyond our physiological means: Small vessel disease of the brain is an expression of a systemic failure in arteriolar function: A unifying hypothesis. Stroke.

[65] Wyss-Coray T., Lin C., Sanan D.A., Mucke L., Masliah E. (2000). Chronic overproduction of transforming growth factor-beta 1 by astrocytes promotes Alzheimer’s disease-like microvascular degeneration in transgenic mice. Am. J. Pathol..

[66] Hamel E. (2015). Cerebral circulation: Function and dysfunction in Alzheimer’s disease. J. Cardiovasc. Pharmacol..

[67] Seo J.H., Maki T., Maeda M., Miyamoto N., Liang A.C., Hayakawa K., Pham L.D., Suwa F., Taguchi A., Matsuyama T. (2014). Oligodendrocyte precursor cells support blood-brain barrier integrity via TGF-beta signaling. PLoS ONE.

[68] Fleenor B.S., Marshall K.D., Durrant J.R., Lesniewski L.A., Seals D.R. (2010). Arterial stiffening with ageing is associated with transforming growth factor-beta1-related changes in adventitial collagen: Reversal by aerobic exercise. J. Physiol..

[69] Meng X.M., Nikolic-Paterson D.J., Lan H.Y. (2016). TGF-beta: The master regulator of fibrosis. Nat. Rev. Nephrol..

[70] Samuel C.S., Hewitson T.D. (2017). Novel therapeutic targets and emerging treatments for fibrosis. Front. Pharmacol..

