# Supplementary material for: MRI Types of Cerebral Small Vessel Disease and Circulating Markers of Vascular Wall Damage
Source: Diagnostics (Basel). 2020 May 29;10(6):354. doi: 10.3390/diagnostics10060354 (PMC7345277; doi:10.3390/diagnostics10060354)
Supplement: Supplementary file 1 [file diagnostics-10-00354-s001.pdf]

Table S1. Severity of WMH in the examined brain regions in patients with cSVD.

WMH, location	Severity on a 4-point scale			
	1 (n, %)	2 (n, %)	3 (n, %)	4 (n, %)
WMH in basal ganglia	48 (50%)	27 (28.1%)	15 (15.6%)	6 (6.3%)
WMH in the anterior region of frontal lobes				
pWMH	4 (4.2%)	43 (44.8%)	34 (35.4%)	15 (15.6%)
dWMH	1 (1%)	38 (39.6%)	42 (43.8%)	15 (15.6%)
jWMH	36 (37.5%)	24 (25%)	17 (17.7%)	19 (19.8%)
WMH in the posterior region of frontal lobes				
pWMH	9 (9.4%)	47 (49%)	24 (25%)	16 (16.7%)
dWMH	0	30 (31.3%)	37 (38.5%)	29 (30.2%)
jWMH	39 (40.6%)	25 (26%)	9 (9.4%)	23 (24%)
WMH in the parietal lobes				
pWMH	20 (20.8%)	32 (33.3%)	20 (20.8%)	24 (25%)
dWMH	2 (2.1%)	27 (28.1%)	33 (34.4%)	34 (35.4%)
jWMH	46 (47.9%)	24 (25%)	11 (11.5%)	15 (15.6%)
WMH in the temporal lobes				
pWMH	59 (61.5%)	23 (24%)	13 (13.5%)	1 (1%)
dWMH	13 (13.5%)	49 (51%)	34 (35.4%)	0
jWMH	52 (54.2%)	24 (25%)	12 (12.5%)	8 (8.3%)
WMH in the occipital lobes				
pWMH	49 (51%)	36 (37.5%)	11 (11.5%)	0
dWMH	50 (52.1%)	36 (37.5%)	10 (10.4%)	0
jWMH	83 (86.5%)	12 (12.5)	1 (1%)	0

pWMH, periventricular WMH; dWMH, deep WMH; jWMH, juxtacortical WMH

Table S2. Location and severity of lacunes in patients with cSVD.

Location	None	Up to 5	From 5 to 10	More than 10
Cerebral white matter	54 (56.3%)	16 (16.7%)	9 (9.4%)	17 (17.7%)
Basal ganglia	64 (66.7%)	11 (11.5%)	9 (9.4%)	12 (12.5%)
Brainstem	69 (71.9%)	19 (19.8%)	8 (8.3%)	0
Cerebellum	78 (81.3%)	10 (10.4%)	5 (5.2%)	3 (3.1%)

Table S3. Location and severity of cerebral microbleeds in patients with cSVD.

Microbleeds	None	Up to 5	From 5 to 10	More than 10
Basal ganglia	68 (70.8%)	12 (12.5%)	5 (5.2%)	11 (11.5%)
WMH in the anterior region of frontal lobes:				
pWMH	96 (100%)	0	0	0
dWMH	92 (95.8%)	4 (4.2%)	0	0
jWMH	75 (78.1%)	12 (12.5%)	5 (5.2%)	4 (4.2%)
WMH in the posterior region of frontal lobes:				

pWMH	95 (99%)	1 (1%)	0	0
dWMH	86 (89.6%)	9 (9.4%)	1 (1%)	0
jWMH	77 (80.2%)	13 (13.5%)	1 (1%)	5 (5.2%)
WMH in the parietal lobes:				
pWMH	95 (99%)	1 (1%)	0	0
dWMH	92 (95.8%)	3 (3.1%)	1 (1%)	0
jWMH	75 (78.1%)	8 (8.3%)	2 (2.1%)	11 (11.5%)
WMH in the temporal lobes:				
pWMH	96 (100%)	0	0	0
dWMH	95 (99%)	1 (1%)	0	0
jWMH	72 (75%)	12 (12.5%)	2 (2.1%)	10 (10.4%)
WMH in the occipital lobes:				
pWMH	96 (100%)	0	0	0
dWMH	96 (100%)	0	0	0
jWMH	79 (82.3%)	11 (11.5%)	4 (4.2%)	2 (2.1%)

pWMH, periventricular WMH; dWMH, deep WMH; jWMH, juxtacortical WMH

Table S4. Widening of subarachnoid spaces in the examined brain regions of patients with cSVD.

**Brain regions**	**No widening**	**Mild widening**	**Moderate widening**	**Severe widening**
Anterior region of the frontal lobes	39 (40.6%)	51 (53.1%)	6 (6.3%)	0
Posterior region of the frontal lobes	47 (49%)	46 (47.9%)	3 (3.1%)	0
Parietal lobes	63 (65.6%)	33 (34.4%)	0	0
Temporal lobes	81 (84.3%)	15 (15.6%)	0	0
Occipital lobes	89 (92.7%)	7 (7.3%)	0	0
